# Improved Magnetization Transfers among Quadrupolar Nuclei in Two-Dimensional Homonuclear Correlation NMR Experiments Applied to Inorganic Network Structures

**DOI:** 10.3390/molecules25020337

**Published:** 2020-01-14

**Authors:** Yang Yu, Philipp Keil, Michael Ryan Hansen, Mattias Edén

**Affiliations:** 1 Department of Materials and Environmental Chemistry, Stockholm University, SE-106 91 Stockholm, Sweden; yang.yu@mmk.su.se; 2 Institute for Physical Chemistry, Westfälische Wilhelms-Universität Münster, DE-48 149 Münster, Germany; philipp.keil@uni-muenster.de (P.K.); mhansen@uni-muenster.de (M.R.H.)

**Keywords:** interatomic connectivities, quadrupolar nuclei, half-integer spins, dipolar recoupling, ^11^B NMR, ^27^Al NMR, microporous aluminophosphate, borosilicate glass, glass structure

## Abstract

We demonstrate that supercycles of previously introduced two-fold symmetry dipolar recoupling schemes may be utilized successfully in homonuclear correlation nuclear magnetic resonance (NMR) spectroscopy for probing proximities among half-integer spin quadrupolar nuclei in network materials undergoing magic-angle-spinning (MAS). These (*S*R2${}_{2}^{1}$)M, (*S*R2${}_{4}^{1}$)M, and (*S*R2${}_{8}^{1}$)*M* recoupling sequences with M=3 and M=4 offer comparably efficient magnetization transfers in single-quantum–single-quantum (1Q–1Q) correlation NMR experiments under moderately fast MAS conditions, as demonstrated at 14.1 T and 24 kHz MAS in the contexts of ${}^{11}$B NMR on a Na${}_{2}$O–CaO–B${}_{2}$O${}_{3}$–SiO${}_{2}$ glass and ${}^{27}$Al NMR on the open framework aluminophosphate AlPO-CJ19 [(NH${}_{4}$)${}_{2}$Al${}_{4}$(PO${}_{4}$)${}_{4}$HPO${}_{4}\cdot$H${}_{2}$O]. Numerically simulated magnetization transfers in spin–3/2 pairs revealed a progressively enhanced tolerance to resonance offsets and rf-amplitude errors of the recoupling pulses along the series (*S*R2${}_{2}^{1}$)M< (*S*R2${}_{4}^{1}$)M< (*S*R2${}_{8}^{1}$)*M* for increasing differences in chemical shifts between the two nuclei. Nonetheless, for scenarios of a relatively minor chemical-shift dispersions (≲3 kHz), the (*S*R2${}_{2}^{1}$)*M* supercycles perform best both experimentally and in simulations.

## 1. Introduction

Magic-angle-spinning (MAS) nuclear magnetic resonance (NMR) spectroscopy offers a complementary structural probe to diffraction techniques, where MAS NMR is particularly powerful for studies of disordered structures [1,2]. Moreover, solid-state NMR may also unveil information across the “difficult” 0.3–1 nm length-scale, unlike other spectroscopies, such as infrared and Raman. Here the through-space mediated *dipolar interaction* constitutes the key NMR tool for the structural probing [1,2,3,4,5,6,7]. The dipolar-interaction strength in a pair of homonuclear spins *j* and *k* is given by the dipolar coupling constant ($b_{jk}$; in Hz), which depends on the inverse cube of the *j*–*k* distance ($r_{jk}$) according to $b_{jk}=-\mu_{0}\hbar \gamma^{2}r_{jk}^{-3}/\left(8\pi^{2}\right)$, where γ is the gyromagnetic ratio [3,4,5]. The utilization of *dipolar recoupling* for retrieving both qualitative information about interatomic proximities and quantitative interatomic distances is nowadays exploited routinely in MAS NMR [1,2,3,4,5,6,7]. Implementations normally require application of dipolar recoupling radio-frequency (rf) pulse sequences to “recouple” (i.e., restore) the dipolar effects (which are otherwise suppressed by MAS) in a controlled fashion [3,4,5,6,7].

The engineering of rf-pulse sequences to achieve homonuclear dipolar recoupling among spins-1/2 (e.g., ${}^{1}$H, ${}^{13}$C, and ${}^{31}$P) is well-developed [3,4,5,6,7], whereas it is exceedingly difficult to devise efficient and robust dipolar recoupling methods for half-integer spin quadrupolar nuclei [8,9], such as the ${}^{11}$B (spin S=3/2) and ${}^{27}$Al (S=5/2) nuclides considered herein. Underlying these problems is the necessity to solely observe and control the central transition (CT) of the quadrupolar nucleus [10], thereby requiring the lowest possible rf-field amplitudes (“rf power”) to avoid NMR-signal leakages out to the satellite transitions (STs) during the dipolar recoupling, while it must also cope with large second-order broadenings (and chemical-shift dispersion for structurally disordered materials [2,10]). See refs. [8,9] for further information.

To recouple the homonuclear dipolar interactions among half-integer spins but avoiding rf-pulse application [11,12,13,14], early work borrowed techniques introduced for spins-1/2, then utilizing rotational resonance [15] and off-MAS, however, with the disadvantages of either compromising the (already limited) NMR spectral resolution, or requiring non-standard MAS probeheads. Another “dipolar recoupling” option exploited in various structural studies [16,17,18,19] is the “spontaneous” reintroduction of the homonuclear dipolar interaction due to its interference with the first-order quadrupolar interaction during MAS (known as “quadrupolar-driven” recoupling [17,20,21,22]), or with heteronuclear dipolar interactions involving ${}^{1}$H (“proton-driven” recoupling [17,23,24,25]).

The first attempts to utilize rf fields for recoupling half-integer spins [26,27,28] involved the 2Q-HORROR technique introduced for spins-1/2 [29]. Its very low nutation frequency (1/2 of the MAS rate) renders this method highly advantageous for minimizing the CT-signal losses under MAS. Here a major achievement was the demonstration by Mali et al. [28] of using 2Q-HORROR for generating two-spin CT double-quantum coherences (2QC) in a single-quantum–double-quantum (1Q–2Q) NMR correlation protocol for probing spatial proximities among half-integer spins. The nowadays routine usage of *multiple*-pulse rf sequences for recoupling quadrupolar nuclei was introduced by Edén et al. [30], who applied MQ-phase cycled [7,31,32,33,34] symmetry-based [6,7] rf-pulse sequences during the mixing periods in 2D correlation NMR protocols; these schemes have been used to recouple homonuclear ${}^{11}$B, ${}^{23}$Na, and ${}^{27}$Al spins in ceramics, minerals, and catalysts [30,35]. Yet, the earlier dipolar recoupling sequences of ref. [30] lead to comparatively large NMR-signal losses, particularly for prolonged rf application (>10 ms) relative to the alternative recoupling options introduced herein.

Site-resolved information about pair-wise spatial proximities may be obtained by using 2D correlation NMR techniques that combine the local structural information from the chemical shifts with dipolar recoupling [2,3,4,5,6,7]. The most straightforward homonuclear correlation MAS NMR experiment relates the chemical shifts (i.e., the 1Q coherences; 1QC) in each spectral dimension [17,23,25]: it will henceforth be termed a single-quantum–single-quantum (1Q–1Q) correlation NMR experiment. The rf pulse scheme is identical to the well-known “NOESY” experiment in solution NMR [36]: it correlates the time-evolution of 1QC in both the indirect ($t_{1}$) and direct ($t_{2}$) dimensions, which are interleaved by a *mixing period* ($\tau_{\mathrm{mix}}$) [4,36] during which the dipolar recoupling sequence is applied, thereby leading to longitudinal (“*z*”) magnetization-exchange processes among spatially proximate spin sites [3,5].

Improved spectral resolution among the quadrupolar-broadened NMR signals is offered by 2Q–1Q NMR correlation protocols [28,37,38], or by integrating the triple-quantum MAS (3QMAS) technique [39] with spontaneous or active dipolar recoupling [24,25,30,40,41]. Other options for improving the spectral resolution involves 6Q–1Q correlations [40,42] or arranging magnetizations transfers driven either by dipolar [18,19,43] or *J* couplings [44] under high-resolution double-rotation (DOR) [45,46] conditions. Owing to the overall low NMR-signal sensitivity in homonuclear correlation NMR experimentation on half-integer spins, its application is in general restricted to 2D NMR, with a few notable exceptions of implementations of 3D NMR correlation protocols, albeit the practical demonstrations were limited to relatively simple model samples [16,47,48].

Herein, we demonstrate that relative to the recoupling options of ref. [30], more efficient magnetization transfers and lower NMR-signal losses are offered in 1Q–1Q correlation NMR experiments by using supercycles of two-fold symmetry recoupling schemes, *S*R2${}_{2^{p}}^{1}$ (see Section 2). These pulse sequences were introduced for two-spin 2QC excitation among the CTs of half-integer spins [9,38,49]. The recoupling schemes have been exploited for probing proximities among homonuclear ${}^{11}$B, ${}^{23}$Na, and ${}^{27}$Al sites in a multitude of structurally well-ordered, as well as disordered, network-based materials [8,9,38,41,49,50,51,52,53,54,55,56]. Moreover, they have also been employed in several structural studies of both organic and inorganic systems where spin-1/2 nuclei were used as probes, e.g., ${}^{13}$C [57,58,59,60], ${}^{1}$H [61,62], and ${}^{31}$P [63,64,65].

The spatial proximity information offered by MAS NMR that incorporates dipolar recoupling is particularly valuable for structurally disordered systems, such as glasses, for which essentially no other experimental techniques may readily provide detailed structural insight into the sub-nanometer scale. Here 2Q–1Q ${}^{27}$Al NMR has been employed to probe proximities among the various Al${}^{\left[4\right]}$ (AlO${}_{4}$), Al${}^{\left[5\right]}$ (AlO${}_{5}$), and Al${}^{\left[6\right]}$ (AlO${}_{6}$) coordinations in glasses, including aluminates [49], aluminophosphates [66], aluminosilicates [54,67], and aluminoborates [68]. Likewise, homonuclear correlation ${}^{11}$B NMR experimentation is reported for borosilicate-based glasses [55,56,69], including the Pyrex composition [70,71].

In this article, we demonstrate the successful application of supercycled dipolar recoupling sequences—denoted (*S*R2${}_{2^{p}}^{1}$)*M* and reviewed in Section 2—during the mixing period of the 1Q–1Q correlation NMR protocol. We provide 2D NMR experiments on a borosilicate glass of molar composition 0.124Na${}_{2}$O–0.124CaO–0.501B${}_{2}$O${}_{3}$–0.251SiO${}_{2}$ (referred to as “NCBS”), as well as on the open framework aluminophosphate AlPO-CJ19 [(NH${}_{4}$)${}_{2}$Al${}_{4}$(PO${}_{4}$)${}_{4}$HPO${}_{4}\cdot$H${}_{2}$O] [72]. Furthermore, for altogether six (*S*R2${}_{2^{p}}^{1}$)3 and (*S*R2${}_{2^{p}}^{1}$)4 supercycles, we evaluate the robustness of the dipolar recoupling to resonance offsets and rf-amplitude mis-settings (“rf inhomogeneity”) by numerical simulations of magnetization transfers in ${}^{23}$Na–${}^{23}$Na pairs.

## 2. Rf Pulse Sequences

### 2.1. 1Q–1Q Correlation Protocol

**Figure 1:**
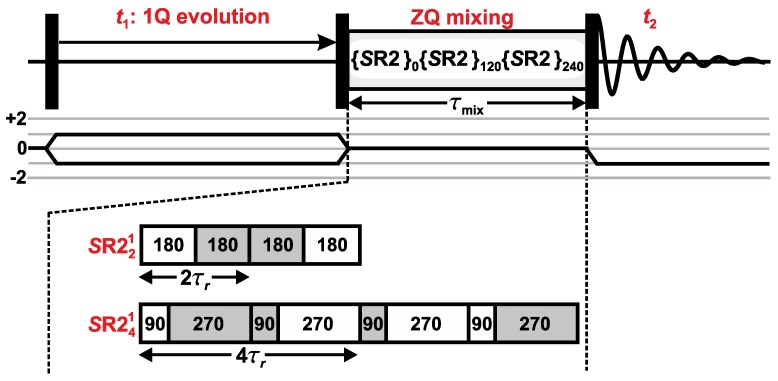
Homonuclear 1Q–1Q NMR correlation rf-pulse protocol involving a mixing period ($\tau_{\mathrm{mix}}$), during which an MQ-phase supercycled *S*R$2_{2^{p}}^{1}$ dipolar recoupling sequence, (*S*R$2_{2^{p}}^{1}$)*M*, is applied to drive transfers of longitudinal central transition (CT) magnetization among proximate half-integer spin quadrupolar nuclei. The black rectangles illustrate CT-selective $90^{\circ }$ pulses. The figure exemplifies the utilization of an (*S*R$2_{2^{p}}^{1}$)3 sequence during $\tau_{\mathrm{mix}}$, i.e., employing M=3 in Equation (1). The *S*R$2_{2}^{1}$ (p=1) and *S*R$2_{4}^{1}$ (p=2) pulse-trains are depicted below the correlation diagram. They operate at the low-power condition $\nu_{\mathrm{nut}}^{\mathrm{CT}}=\nu_{r}/2$; each pulse is illustrated by a box specifying the flip angle (in degrees), whereas white and gray color indicate rf phases of $90^{\circ }$ and $270^{\circ }$, respectively.

Figure 1 illustrates the prototype rf-pulse protocol for homonuclear 1Q–1Q NMR correlations among half-integer spin quadrupolar nuclei by employing a dipolar recoupling scheme for driving longitudinal magnetization-exchange processes during the mixing period [30]. Note that all rf pulses are CT selective. Herein we consider recoupling sequences that provide a “ZQ effective Hamiltonian”, meaning that it involves $S_{j}^{+}S_{k}^{-}$ and $S_{j}^{-}S_{k}^{+}$ “flip-flop” operators for two recoupled spins *j* and *k* [3,4,5,6,7]. Such a pulse sequence provides a magnetization transfer from spin *j*, resonating at $\nu_{j}$, to another nearby spin-site *k* that resonates at $\nu_{k}$. This j→k magnetization transfer is reflected by a *cross peak* centered at the 2D-frequency coordinate {$\nu_{1},$$\nu_{2}\}=\{\nu_{j},$ $\nu_{k}$} in the 1Q–1Q NMR spectrum. Likewise, the k→j magnetization-transfer process yields a cross peak at {$\nu_{1},$$\nu_{2}\}=\{\nu_{k},$$\nu_{j}$}. Then in the absence of resonance-broadenings from second-order quadrupolar interactions and (potential) chemical-shift dispersions from structural disorder, the 1Q–1Q correlation NMR spectrum manifests two narrow cross peaks from the *j*–*k* spin-pair. In practice, however, distributions of resonances around each $\nu_{j}$ and $\nu_{k}$ frequency value produce broad “ridge-like” cross peaks in the 2D NMR spectrum [8,9].

### 2.2. (SR$2_{2^{p}}^{1})M$ Supercycles for Magnetization Exchange

The pulse schemes used herein for “ZQ recoupling” build on MQ-phase cycles [7,30,31,32,33,34,73], denoted (S)*M* in the nomenclature of refs. [7,32,34], where S is a dipolar recoupling rf-pulse sequence. The MQ-phase supercycle involves a concatenation of *M* pulse-trains S, each being successively phase-shifted by $360^{\circ }/M$ (i.e., by 2π/M radians) relative to the previous one:

(1) $$\left(S\right)M\equiv {\left\{S\right\}}_{0}{\left\{S\right\}}_{360/M}{\left\{S\right\}}_{720/M}...{\left\{S\right\}}_{360(M-1)/M}.$$

Here ${\left\{\cdots \right\}}_{\phi }$ implies a phase-shift by ϕ of all pulses within the braces. We note that the MQ-phase-cycle notation originally introduced in refs. [32,34] involved a superscript of *M* (e.g., $M^{1}$) that for simplicity is omitted herein.

In ref. [30], Edén et al. introduced M=3 and M= 4 supercycles in the context of recoupling half-integer spins, utilizing the symmetry-based [6,7] pulse sequence *S*R$4_{4}^{1}\equiv$ R$4_{4}^{1}$R$4_{4}^{-1}$ [33,74]. It is based on a “windowed” rf-pulse element (τ–$180^{\circ }$–τ) with duration of one rotor period $\tau_{r}=\nu_{r}^{-1}$, where $\nu_{r}$ is the MAS rate in Hz. The element involves a weak $180^{\circ }$ pulse, whose duration $\tau_{180}$ spans only a fraction $f_{180}=\tau_{180}$/$\tau_{r}$ of $\tau_{r}$. Such “windowed” *S*R$4_{4}^{1}$ schemes were originally developed for dipolar recoupling of spins-1/2 by Ishii [74], who called the pulse-sequences “finite-pulse RFDR” (whereas the original RFDR scheme involves strong pulses [75]). As will be shown herein and discussed further in an upcoming paper, a significantly improved recoupling performance is accomplished by instead utilizing two-fold symmetry schemes, *S*R$2_{2^{p}}^{1}$ [9,38,49,57,58] as the basic pulse-train S in the MQ phase cycle (Equation (1)). The *S*R$2_{2^{p}}^{1}$ sequences were initially designed for usage in 2Q–1Q correlation NMR protocols [38,41,49].

The family of *S*R$2_{2^{p}}^{1}$ sequences with p={1,2,3,...} is generated recursively by combining cyclic pulse-permutations and phase-inversions, as described in detail in refs. [9,57,58]. For increasing *p*, the compensation to resonance offsets and rf-amplitude errors enhances for the *S*R$2_{2^{p}}^{1}$ sequence [9,57,58], as demonstrated further in Section 4.1. Each rotor-synchronized *S*R$2_{2^{p}}^{1}$ member spans $2^{p+1}$ rotor periods and operates at the low-power 2Q–HORROR condition [29], meaning that the CT nutation frequency is $\nu_{\mathrm{nut}}^{\mathrm{CT}}=\left(S+1/2\right)\gamma B_{1}=\left(S+1/2\right)\nu_{\mathrm{nut}}=\nu_{r}/2$ [9,57,58], where $B_{1}$ is the rf-field amplitude.

Any *S*R$2_{2^{p}}^{1}$ rf-pulse sequence is formed by merging two pulse-blocks, R$2_{2^{p}}^{1}$ and R$2_{2^{p}}^{-1}$, the latter obtained by sign-reversal of all rf-phases of R$2_{2^{p}}^{1}$ [7,33,34]. Onwards, we only consider the first three *S*R$2_{2^{p}}^{1}$ members (see ref. [58]) and employ the shorthand notation *S*R2${}_{2}^{1}\equiv$R$2_{2}^{1}$R$2_{2}^{-1}$ (p=1), *S*R2${}_{4}^{1}\equiv$R$2_{4}^{1}$R$2_{4}^{-1}$ (p=2), and *S*R2${}_{8}^{1}\equiv$R$2_{8}^{1}$R$2_{8}^{-1}$ (p=3). In explicit rf-pulse nomenclature $\beta_{\phi }$ (where β and ϕ denote the flip angle and phase of the rf pulse, respectively), these schemes correspond to

(2) $$SR2_{2}^{1}\equiv R2_{2}^{1}R2_{2}^{-1}\equiv 180_{90}360_{270}180_{90}$$

(3) $$SR2_{4}^{1}\equiv R2_{4}^{1}R2_{4}^{-1}\equiv 90_{90}360_{270}270_{90}90_{270}360_{90}270_{270}$$

(4) $$SR2_{8}^{1}\equiv R2_{8}^{1}R2_{8}^{-1}\equiv 360_{270}270_{90}90_{270}360_{90}270_{270}450_{90}270_{270}90_{90}360_{270}270_{90}90_{270}$$

where the first two members are illustrated in Figure 1.

The combination of a *S*R$2_{2^{p}}^{1}$ scheme and an MQ-phase cycle yields a supercycle (*S*R$2_{2^{p}}^{1}$)*M* that spans $2^{p+1}M$ rotor periods. Herein, we employ either M=3—which involves consecutive phase-shifts of {0,$120^{\circ },$$240^{\circ }\}$ [32,33,34]—or M=4 that implies the sequence of phase shifts {0,$90^{\circ },$$180^{\circ },$$270^{\circ }\}$ [30]. Consequently, if (for instance) *S*R2${}_{2}^{1}$ is utilized in an (S)3 supercycle, Equation (1) yields the following explicit pulse train:

(5)(SR221)3≡{SR221}0{SR221}120{SR221}240(6)=180903602701809018021036030180210180330360150180330,

whereas the (SR$2_{2}^{1})4$ counterpart becomes

(7)(SR221)4≡{SR221}0{SR221}90{SR221}180{SR221}270(8)=180903602701809018018036001801801802703609018027018003601801800.

As discussed further in refs. [9,38,58], all *S*R$2_{2^{p}}^{1}$ schemes give “mixed ZQ/2Q recoupling”, meaning that the effective dipolar Hamiltonian comprises *both* 2Q ($S_{j}^{+}S_{k}^{+}$ and $S_{j}^{-}S_{k}^{-}$) and ZQ ($S_{j}^{+}S_{k}^{-}$ and $S_{j}^{-}S_{k}^{+}$) spin operators. As these ZQ/2Q operators interfere destructively in multi-spin systems [58,76], it is necessary to arrange *pure* 2Q *or* ZQ recoupling. The former is accomplished by sandwiching the *S*R$2_{2^{p}}^{1}$ pulse train between two CT-selective $90^{\circ }$ pulses and is denoted [*S*R$2_{2^{p}}^{1}$] [38,58], while ZQ recoupling is achieved by using an MQ phase-cycle with M⩾3 [7,30,33,58]. The effective dipolar Hamiltonian is identical for all (*S*R$2_{2^{p}}^{1}$)*M* schemes; see refs. [9,58] for further information and explicit Hamiltonian expressions.

## 3. Materials and Methods

### 3.1. Samples

A 6.0 g batch of the NCBS glass of molar composition 0.124Na${}_{2}$O–0.124CaO–0.501B${}_{2}$O${}_{3}$–0.251SiO${}_{2}$ was prepared by a traditional melt-quench technique. Precursors of SiO${}_{2}$ (99.99%), Na${}_{2}$CO${}_{3}$ (99.5%), and CaCO${}_{3}$ (99%) from ChemPur, and H${}_{3}$BO${}_{3}$ (99.9%) from Sigma, were mixed in a mortar. The mixture was transferred to a Pt crucible and decarbonated by heating in an electric furnace at 950 ${}^{\circ }$C for 2 h. The temperature was then raised to 1150 ${}^{\circ }$C and the melt was held for 20 min, after which it was quenched by immersing the bottom of the crucible in water. The B${}_{2}$O${}_{3}$ content was determined to be 54.0 wt% by ${}^{11}$B MAS NMR, which is in excellent agreement with the nominal value 53.9 wt% (i.e., 0.2% relative discrepancy) [56]. Hence, given that B is the most volatile element in the melt, we may safely assume that the nominal elemental batch composition is representative for the glass.

The AlPO-CJ19 sample [(NH${}_{4}$)${}_{2}$Al${}_{4}$(PO${}_{4}$)${}_{4}$HPO${}_{4}\cdot$H${}_{2}$O] was prepared as described in ref. [72] and was kindly provided by Dan Zhou and Jihong Yu at Jilin University (P.R. China). Dipolar recoupling applications are reported previously on the same sample [38,49,76].

### 3.2. Solid-State NMR Experiments

The ${}^{11}$B (S=3/2) and ${}^{27}$Al (S=5/2) NMR experimentation was performed with a Bruker Avance-III spectrometer (Bruker BioSpin, Rheinstetten, Germany) at the magnetic field $B_{0}$=14.1 T, which gives ${}^{11}$B and ${}^{27}$Al Larmor frequencies of −192.5 MHz and −156.37 MHz, respectively. Powders of NCBS and AlPO-CJ19 were filled in 3.2 mm zirconia rotors and spun at $\nu_{r}=24.00$ kHz. Neat BF${}_{3}\cdot$OEt${}_{2}$ and a 1 M Al(NO${}_{3}$)${}_{3}$ aqueous solution were used for ${}^{11}$B and ${}^{27}$Al shift referencing, respectively, as well as for determining the nutation frequencies for ${}^{11}$B ($\nu_{B}$) and ${}^{27}$Al ($\nu_{\mathrm{Al}}$) of all *strong* rf pulses. Note that nearly all parts of the experiments involved CT-selective pulses, where the CT nutation frequency is given by $\nu_{E}^{\mathrm{CT}}\approx \left(S+1/2\right)\nu_{E}$, with E={Al, B} [10].

Resonance offsets were minimized by positioning the rf carrier ($\delta_{\mathrm{rf}}$) at the mid-point shift of the ${}^{11}$B/${}^{27}$Al NMR signal region, except during the dipolar recoupling rf-pulses (see below). To accomplish absorptive 2D NMR peaks with frequency-sign discrimination along the indirect spectral dimension, all 2D NMR acquisitions implemented the States-TPPI procedure [77]. Note that the number of $t_{1}$ increments stated below refers to that collected for *each* real and imaginary data-set of the hypercomplex protocol.

#### 3.2.1. ${}^{11}$B MAS NMR on the NCBS Glass

A ${}^{11}$B MAS NMR spectrum was recorded from the NCBS glass by employing short and strong rf pulses (0.33 μs; $13^{\circ }$ flip angle) operating at $\nu_{B}=105$ kHz, using 15 s relaxation delays and 512 accumulated signal transients. The fractional populations {$x_{B}^{\left[3\right]}=0.58$, $x_{B}^{\left[4\right]}=0.42$} of the co-existing {B${}^{\left[3\right]}$, B${}^{\left[4\right]}$} coordinations in the glass were extracted from the ${}^{11}$B MAS NMR spectrum by using the procedure of Massiot et al. [78], as described in ref. [56]. The MAS NMR spectrum was also exploited for estimating the average values of the isotropic chemical shifts (${\bar{\delta }}_{\mathrm{iso}}^{\left[3\right]}=17.4$ ppm and ${\bar{\delta }}_{\mathrm{iso}}^{\left[4\right]}=0.4$ ppm) and quadrupolar products (${\bar{C}}_{Q\eta }^{\left[3\right]}=2.66$ MHz and ${\bar{C}}_{Q\eta }^{\left[4\right]}=0.44$ MHz) of the respective ${}^{11}$B${}^{\left[3\right]}$ and ${}^{11}$B${}^{\left[4\right]}$ sites by employing the protocol described in refs. [10,79]. Here $C_{Q\eta }=C_{Q}\sqrt{1+\eta^{2}/3}$, where $C_{Q}=e^{2}qQ/h$ and η is the quadrupolar coupling constant (in Hz) and the asymmetry parameter of the electric-field gradient (efg) tensor, respectively [10].

The 1Q–1Q correlation ${}^{11}$B NMR spectra from the NCBS glass were recorded with the rf-pulse protocol of Figure 1, using the (SR$2_{2}^{1})4$, (SR$2_{4}^{1})4$ or (SR$2_{8}^{1})4$ supercycles for variable mixing periods of $\tau_{\mathrm{mix}}=\{1.33$, 5.33, 10.67} ms, except for the shortest excitation period for (*S*R$2_{8}^{1})4$, which was $\tau_{\mathrm{mix}}=2.67$ ms. The shortest values of $\tau_{\mathrm{mix}}$ correspond to two completed (*S*R2${}_{2}^{1}$)4 sequences, and one completed (*S*R2${}_{4}^{1}$)4 and (*S*R2${}_{8}^{1}$)4 scheme. At the longest mixing period, {16, 8, 4} repetitions of the {(*S*R2${}_{2}^{1}$)4, (*S*R2${}_{4}^{1}$)4, (*S*R2${}_{8}^{1}$)4} schemes were utilized. The recoupling pulses operated at the ${}^{11}$B CT nutation frequency $\nu_{B}^{\mathrm{CT}}=\nu_{r}/2=12.0$ kHz, whereas the CT-selective $90^{\circ }$ pulses of duration 13.5 μs operated at $\nu_{B}^{\mathrm{CT}}=18.5$ kHz. The rf carrier was set at $\delta_{\mathrm{rf}}=10.4$ ppm (Ω=-32 Hz), $\delta_{\mathrm{rf}}=3.4$ ppm (Ω=1318 Hz), and $\delta_{\mathrm{rf}}=2.6$ ppm (Ω=1468 Hz) during the (*S*R$2_{2}^{1})4$, (*S*R$2_{4}^{1})$4, and (*S*R$2_{8}^{1})$4 rf pulses, respectively, where the numbers in parentheses specify the frequency offset relative to the center-of-gravity frequency (which defines Ω=0) of a CT-selective ${}^{11}$B MAS NMR spectrum recorded under otherwise identical conditions. At the start of each transient of the 1Q–1Q correlation NMR experiment, a WURST pulse [80,81] of duration $\tau_{\mathrm{WURST}}=1.00$ ms ($\nu_{B}^{\mathrm{CT}}=25$ kHz) was applied to enhance the CT-signal intensity [40]. The frequency of the pulse was swept by ±12 kHz around Ω=120 kHz. This provided 2.4 and 1.9 stronger NMR-signal intensities from the ${}^{11}$B${}^{\left[3\right]}$ and ${}^{11}$B${}^{\left[4\right]}$ sites, respectively, and resulting in “apparent” fractional populations of {$x_{B}^{\left[3\right]}=0.64$, $x_{B}^{\left[4\right]}=0.36$}. 120($t_{1}$) × 3989($t_{2}$) time-points were recorded with dwell times {$\Delta t_{1}=4\tau_{r}$, $\Delta t_{2}=5.0$ μs}, 32 accumulated signal transients per $t_{1}$-value and 1.0 s relaxation delays. Although the 2D NMR experimentation required short relaxation delays for reducing the experimental time, the relative ${}^{11}$B${}^{\left[3\right]}$ and ${}^{11}$B${}^{\left[4\right]}$ NMR signal intensities adequately reproduced the corresponding site population in the glass. The data set was zero-filled to 256 × 16,384 points before Fourier transformation.

**Figure 2:**
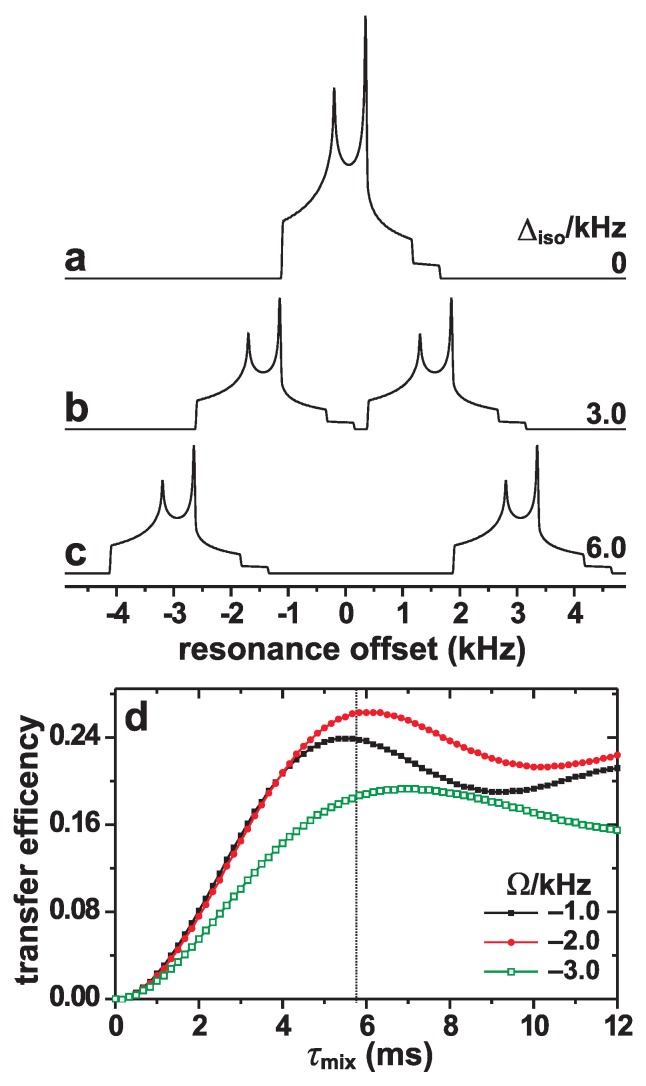
(**a**) Numerically simulated NMR spectra for a powder of two dipolar-coupled spins-3/2 at $B_{0}=14.1$ T and $\nu_{r}=24.00$ kHz MAS. The spin-system parameters in (**a**) correspond to those of two ${}^{23}$Na sites in Na${}_{2}$SO${}_{4}$, which were taken from ref. [92]: $\Delta_{\mathrm{iso}}=0$; $b_{jk}=-259$ Hz; $C_{Q}^{j}=C_{Q}^{k}=2.60$ MHz; $\eta^{j}=\eta^{k}=0.6$, except for the orientations of the three perpendicular dipolar and efg tensors, expressed by the respective Euler angles $\Omega_{D}=\left\{0,0,0\right\}$, $\Omega_{Q}^{j}=\left\{0,90^{\circ },0\right\}$, and $\Omega_{Q}^{k}=\left\{0,90^{\circ },90^{\circ }\right\}$. (**b**,**c**) As in (**a**), but for isotropic chemical shift differences of (**b**) $\Delta_{\mathrm{iso}}=3.0$ kHz and (**c**) $\Delta_{\mathrm{iso}}=6.0$ kHz. (**d**) Simulated magnetization transfer efficiencies—i.e., the fraction of longitudinal CT-magnetization of spin-site *j* transferred to spin *k*—for an increasing mixing period ($\tau_{\mathrm{mix}}$) of (*S*R2${}_{2}^{1}$)4 dipolar-recoupling application. The curves were calculated for $\Delta_{\mathrm{iso}}=0$ and resonance offsets Ω={-3.0,-2.0, -1.0} kHz. The dotted line at $\tau_{\mathrm{mix}}=6.00$ ms marks the mixing period used to evaluate the results of Figure 3 and Figure 4 for the case of the (*S*R2${}_{2}^{1}$)4 supercycle.

A 2Q–1Q correlation ${}^{11}$B NMR spectrum was recorded by the rf-pulse scheme depicted in Figure 2d of Edén [8], using a short 2QC excitation period to ensure 2D NMR signal intensities proportional to the respective ${}^{11}$B${}^{\left[p\right]}$–${}^{11}$B${}^{\left[q\right]}$ pair populations, as discussed in detail in ref. [56]. One completed [*S*R2${}_{2}^{1}$] dipolar recoupling sequence [38] was employed for excitation of two-spin CT 2QC, using equal 2QC excitation and reconversion intervals of $\tau_{\mathrm{exc}}=4\tau_{r}=167$ μs. Here the brackets [⋯] imply sandwiching the *S*R2${}_{2}^{1}$ pulse sequence by two CT-selective 90${}^{\circ }$ pulses [38,58]. The rf carrier was set at $\delta_{\mathrm{rf}}=8.4$ ppm for the recoupling pulses. A Hahn-echo of duration $2\tau_{r}$ was applied before the $t_{1}$-evolution period to accomplish rotor-synchronized 2QC excitation and reconversion stages [38], as well as suppression of all undesirable single-spin 2QC associated with the satellite transitions [28]; this was ensured by an 8-step phase cycle of the CT-selective $180^{\circ }$ pulse [41], which was of duration 34.2 μs. The details of the phase-cycle implementation is given in the Supporting Information of ref. [41]. 23($t_{1}$)×600($t_{2}$) time-points were recorded with dwell times {$\Delta t_{1}=2\tau_{r}$, $\Delta t_{2}=8.4$ μs}, 768 accumulated signal transients/$t_{1}$-value and relaxation delays of 1.5 s. The data set was zero-filled to 128 × 8192 points prior to Fourier transformation. Other conditions were as described for the 1Q–1Q correlation NMR experiments, except that no signal enhancement (i.e., WURST) was applied to avoid perturbing the relative 2D NMR signal intensities.

#### 3.2.2. ${}^{27}$Al NMR on AlPO-CJ19

A 1Q–1Q correlation ${}^{27}$Al NMR spectrum was recorded from the AlPO–CJ19 sample with the (SR$2_{4}^{1})3$ scheme for $\tau_{\mathrm{mix}}=30.0$ ms. The recoupling pulses operated near the ${}^{27}$Al CT nutation frequency $\nu_{\mathrm{Al}}^{\mathrm{CT}}=12$ kHz, whereas the CT-selective $90^{\circ }$ pulses of duration 13.0 μs operated at $\nu_{\mathrm{Al}}^{\mathrm{CT}}=19$ kHz. The rf carrier was set at $\delta_{\mathrm{rf}}=12.5$ ppm (Ω=258 Hz) during the recoupling pulses. The parameters for the WURST pulse were as for the 1Q–1Q ${}^{11}$B NMR experiments, except for $\nu_{\mathrm{Al}}=48$ kHz. These conditions resulted in NMR-signal enhancement factors of 2.5, 3.0, and 2.4 for the Al${}^{\left[4\right]}$, Al${}^{\left[5\right]},$ and Al${}^{\left[6\right]}$ sites, respectively. 45($t_{1}$) × 6000($t_{2}$) time-points were recorded with dwell times {$\Delta t_{1}=2\tau_{r}$, $\Delta t_{2}=5.00$ μs}, 1024 accumulated signal transients/$t_{1}$-value and 1.0 s relaxation delays. The data set was zero-filled to 256 × 16,384 points before Fourier transformation.

### 3.3. Numerical Simulations

The numerical simulations were performed with the SIMPSON package (version 4.2.1) [82,83], employing a small-step (<1 μs) integration of the Schrödinger equation [84] during each periodically repeated (*S*R$2_{2^{p}}^{1}$)*M* sequence throughout the mixing interval. The magnetization-transfer efficiency was calculated as the fraction of longitudinal CT magnetization of spin *j* that was transferred to spin *k* ($S_{jz}^{\mathrm{CT}}\to S_{kz}^{\mathrm{CT}}$) within a pair of S=3/2, and was sampled at each completed *S*R$2_{2^{p}}^{1}$ pulse-element of the (*S*R$2_{2^{p}}^{1}$)*M* supercycle out to $\tau_{\mathrm{mix}}$ ≲10 ms. The simulations accounted for all relevant spin-system parameters, i.e., the isotropic chemical shifts, dipolar, and first- as well as second-order quadrupolar interactions, which were typical for ${}^{23}$Na; see the caption to Figure 2. Powder averaging [85] was performed using 3722 three-angle ZCW orientations [86,87]. The corresponding MAS NMR spectra of the two coupled S=3/2 were calculated using the COMPUTE protocol [88,89] and employing the FWTASG spectral interpolation [90] with the ${}^{\mathrm{ROSE}}$LEBh6535 set of orientations [91] for efficient powder averaging. Only the CT signals were detected.

## 4. Results and Discussion

### 4.1. Numerically Simulated Magnetization-Transfer Efficiencies

The very low rf-power requirement of the (SR$2_{2^{p}}^{1})M$ dipolar recoupling schemes (see Section 2) ensures minimal CT-signal leakages to the STs, but compromises the robustness of the recoupling to variations in *resonance offsets* ($\Omega_{j}=\nu_{\mathrm{iso}}^{j}-\nu_{\mathrm{rf}}$) among the various nuclei in the sample. For quadrupolar nuclei, resonance offsets may originate from two sources: (*i*) Distinct isotropic chemical shifts, $\Delta_{\mathrm{iso}}=\nu_{\mathrm{iso}}^{j}-\nu_{\mathrm{iso}}^{k}$, where $\nu_{\mathrm{iso}}^{j}$ ($\nu_{\mathrm{iso}}^{k}$) is the isotropic chemical shift of the spin-site *j* (*k*), and $\nu_{\mathrm{rf}}$ is the rf-carrier frequency. (*ii*) The second-order isotropic quadrupolar shift and the accompanying anisotropic resonance-*broadening* [10], where the latter presents the major obstacle; see the MAS NMR spectra in Figure 2a–c.

The dipolar recoupling must also be robust to spreads of spin nutation frequencies across the sample (“rf inhomogeneity”). The impact of rf inhomogeneity may be gauged from experiments and simulations where the applied rf-amplitude is deliberately mis-set, such that the actual CT nutation frequency during the recoupling pulses ($\nu_{\mathrm{nut}}^{\mathrm{CT}}$) deviates from the *nominal* value $\nu_{\mathrm{nut}}^{\mathrm{CT}}($nom), which for all (SR$2_{2}^{1})M$ schemes obeys $\nu_{\mathrm{nut}}^{\mathrm{CT}}($nom$)=\nu_{r}/2$.

In the following, we evaluate the alterations observed in numerically simulated *magnetization-transfer efficiencies* for variations in either the resonance offset or the *relative* nutation frequency, $\nu_{\mathrm{nut}}^{\mathrm{CT}}/\nu_{\mathrm{nut}}^{\mathrm{CT}}($nom). The transfer efficiency corresponds to the fraction of longitudinal CT-magnetization of spin *j* transferred to spin *k* during a given mixing period ($\tau_{\mathrm{mix}}$). Figure 2d plots examples of magnetization-transfer efficiency curves for increasing $\tau_{\mathrm{mix}}$-values observed for the (*S*R2${}_{2}^{1}$)4 scheme under the conditions described in the caption to Figure 2a. Each transfer efficiency was sampled at each completed *S*R$2_{2}^{1}$ sequence of the (SR$2_{2}^{1}$)4 supercycle.

#### 4.1.1. Resonance Offsets

**Figure 3:**
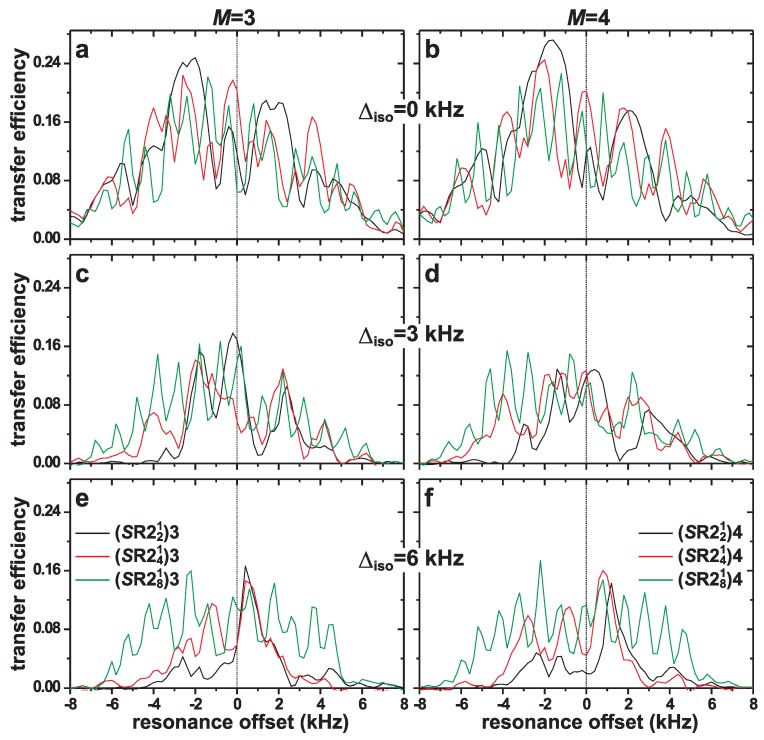
Numerically simulated magnetization-transfer efficiencies plotted against the resonance offset of the (*S*R2${}_{2}^{1}$)*M*, (*S*R2${}_{4}^{1}$)Mand (*S*R2${}_{8}^{1}$)*M* sequences [identified in the legends of (**e**,**f**)] at $B_{0}=14.1$ T and 24.00 kHz MAS. The left and right panels display the results for M=3 and M=4, respectively. All simulations employed a nominal CT nutation frequency during recoupling ($\nu_{\mathrm{nut}}^{\mathrm{CT}}=\nu_{r}/2=12.0$ kHz) and the spin-system parameters of Figure 2 with isotropic chemical shift differences of (**a**,**b**) $\Delta_{\mathrm{iso}}=0$; (**c**,**d**) $\Delta_{\mathrm{iso}}=3.0$ kHz, and (**e**,**f**) $\Delta_{\mathrm{iso}}=6.0$ kHz.

Figure 3 shows numerically simulated magnetization-transfer efficiencies plotted against the resonance offset for various spin-3/2 pairs undergoing 24.00 kHz MAS at $B_{0}=14.1$ T. The corresponding MAS NMR spectra are presented in Figure 2a–c. The (*S*R2${}_{2}^{1}$)*M*, (*S*R2${}_{4}^{1}$)Mand (*S*R2${}_{8}^{1}$)*M* sequences with M=3 and M=4 were evaluated, whose results are presented in the left and right panels of Figure 3, respectively. Here and in the following evaluations, a “zero resonance offset” (Ω=0) implies that the rf carrier frequency coincides with the center-of-gravity frequency of the NMR spectrum [see Figure 2a–c]; the NMR frequency separation between the center-of-gravities of the two powder lineshapes of spins *j* and *k* is then given by $\Delta_{\mathrm{iso}}$ in Figure 2 and Figure 3.

All magnetization-transfer curves of Figure 3 reveal an oscillatory response when the resonance offset varies. Disregarding those undesirable oscillations that are discussed below, the bandwidth across which decent magnetization transfers are observed is markedly increased along the series (*S*R2${}_{2}^{1}$)*M*<(*S*R2${}_{4}^{1}$)*M*<(*S*R2${}_{8}^{1}$)*M* for each fixed value of *M*. This observation accords with previous inferences from simulations and experiments of double-quantum filtration (2QF) responses for both spins-1/2 [57,58] and half-integer spins [49,76], as well as from simulated magnetization transfers in spin-1/2 pairs [58]. Notably, while the construction of the most robust pulse scheme *S*R2${}_{8}^{1}$ was outlined in ref. [76], it has hitherto not been evaluated in the context of half-integer spins.

For a small isotropic chemical-shift dispersion ($\Delta_{\mathrm{iso}}≲3$ kHz), Figure 3a,b evidences that the more complex (*S*R2${}_{4}^{1}$)*M* and (*S*R2${}_{8}^{1}$)*M* schemes offer no advantages of their (*S*R2${}_{2}^{1}$)*M* counterparts, while the results for moderately large shift differences ($3≲\Delta_{\mathrm{iso}}$/kHz ≲6) shown in Figure 3c–f reveal a significantly higher robustness of the (*S*R2${}_{4}^{1}$)*M*—and notably (*S*R2${}_{8}^{1}$)*M*—sequences towards the precise rf-carrier frequency position. At the largest evaluated shift-difference of $\Delta_{\mathrm{iso}}=6$ kHz, the advantages of the (*S*R2${}_{8}^{1}$)3 and (*S*R2${}_{8}^{1}$)4 supercycles are particularly evident.

Concerning the precise selection of MQ-phase cycle [i.e., choice of *M* in Equation 1], no conclusive recommendation may be made from the simulations of Figure 3 alone, as both the (*S*R$2_{2^{p}}^{1}$)3 and (*S*R$2_{2^{p}}^{1}$)4 options differ in details but offer very similar resonance-offset bandwidths for a given *S*R$2_{2^{p}}^{1}$ scheme. Experimentally observed magnetization-transfer efficiencies are also similar for the (*S*R$2_{2^{p}}^{1}$)3 and (*S*R$2_{2^{p}}^{1}$)4 schemes (to be discussed elsewhere), in accordance with the simulated results of Figure 3. Moreover, the (SR$2_{2^{p}}^{1})M$ supercycles employed herein generally offer better spectral signal-to-noise (S/N) ratios relative to their windowed (*S*R4${}_{4}^{1}$)*M* counterparts utilized in ref. [30]; see Section 4.2.3.

The primary weakness with the (*S*R$2_{2^{p}}^{1}$)*M* dipolar recoupling schemes is the strong oscillations observed even for small variations in the precise rf-carrier position (Figure 3). This feature is a clear disadvantage in 1Q–1Q NMR correlation applications for multi-site structures because the cross-peak intensities may not in general be translated into reliable (relative) internuclear distances among the various spin-pairs in the structure, as is possible for spin-1/2 implementation of the (*S*R$2_{2^{p}}^{1})M$ sequences and other homonuclear recoupling options [3,5,62]. As will be demonstrated and discussed further in an upcoming paper, however, the undesirable property of offset-dependent magnetization transfers appears to be a quite general feature and is by no means specific for the (*S*R$2_{2^{p}}^{1}$)*M* recoupling sequences. This observation underscores the difficulties in devising robust homonuclear dipolar recoupling sequences for half-integer spins.

The underlying reasons for the strong resonance-offset dependent oscillations are not known, but similar trends as those observed herein for magnetization transfers when using (*S*R$2_{2^{p}}^{1}$)*M* schemes for ZQ mixing (Figure 3) are also present in previous evaluations of 2QF responses when varying the resonance offset of the [*S*R2${}_{2}^{1}$] and [*S*R2${}_{4}^{1}$] recoupling pulses in 2Q–1Q NMR correlation experiments [49]. Yet, considering the absence of oscillations in *both* magnetization-transfer and 2QF processes for spins-1/2 under otherwise comparable conditions and recoupling sequences [57,58,63], it is evident that the resonance-offset dependent oscillations must stem from interferences between the (very substantial) first-order quadrupolar interactions and the rf pulses. Indeed, the results of Figure 3 suggest that the oscillatory responses versus the resonance offset correlate with the pulse-sequence order *p* of the (*S*R$2_{2^{p}}^{1}$)*M* (see Section 2), meaning that the more complex the pulse train, the faster/more pronounced the oscillations. Hence, they increase in the order (*S*R2${}_{2}^{1}$)*M*<(*S*R2${}_{4}^{1}$)*M*<(*S*R2${}_{8}^{1}$)*M*, which unfortunately partially spoils the advantages of the otherwise *overall* more robust (*S*R2${}_{4}^{1}$)*M* and (*S*R2${}_{8}^{1}$)*M* dipolar recoupling schemes. Such effects might also explain the rather mediocre experimental performance of the (*S*R2${}_{8}^{1}$)*M* schemes when applied to the NCBS glass (Section 4.2).

#### 4.1.2. RF-Amplitude Errors

**Figure 4:**
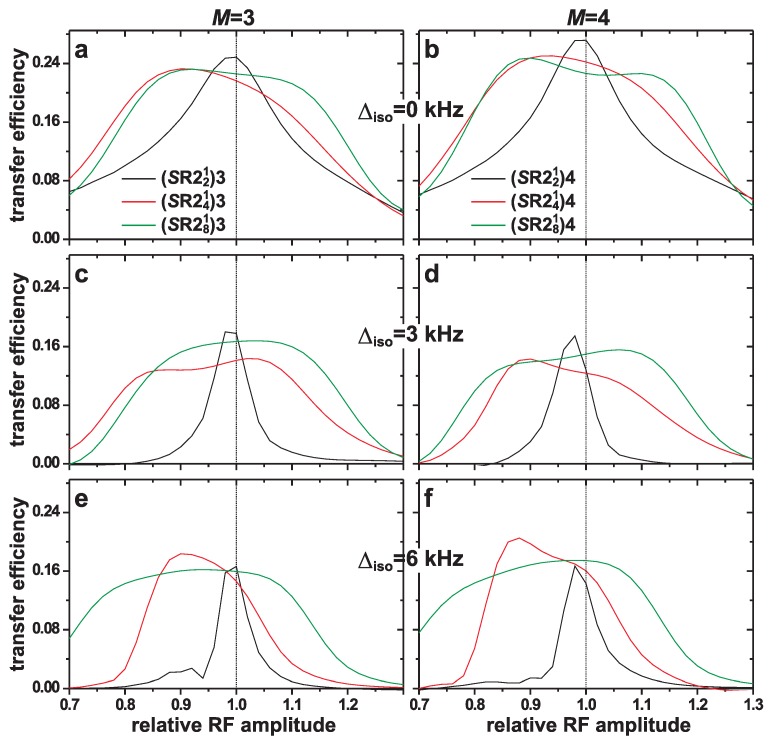
Numerically simulated magnetization transfer efficiencies plotted against the resonance offset of the (*S*R2${}_{2}^{1}$)*M*, (*S*R2${}_{4}^{1}$)Mand (*S*R2${}_{8}^{1}$)*M* sequences [identified in the legends of (**a**,**b**)] at $B_{0}=14.1$ T and 24.00 kHz MAS. The left and right panels display the results for M=3 and M=4, respectively. The simulations employed the spin-system parameters of Figure 2 with isotropic chemical shift differences of (**a**,**b**) $\Delta_{\mathrm{iso}}=0$, (**c**,**d**) $\Delta_{\mathrm{iso}}=3.0$ kHz, and (**e**,**f**) $\Delta_{\mathrm{iso}}=6.0$ kHz.

Figure 4 presents the evaluations of the robustness of each (*S*R2${}_{2}^{1}$)*M*, (*S*R2${}_{4}^{1}$)*M*, and (*S*R2${}_{8}^{1}$)*M* recoupling scheme to rf-amplitude mis-settings from the nominal value $\nu_{\mathrm{nut}}^{\mathrm{CT}}=\nu_{r}/2$. Using the conditions and parameters as in Figure 3, each magnetization-transfer efficiency curve was evaluated at the optimal resonance-offset value but with the (relative) nutation frequency of the CT varied. As for the resonance offset compensation, the results of Figure 4 reveal a progressively enhanced robustness to rf-amplitude errors along the series (*S*R2${}_{2}^{1}$)*M*<(*S*R2${}_{4}^{1}$)*M*<(*S*R2${}_{8}^{1}$)*M*, whereas no significant differences are observed among each (*S*R$2_{2^{p}}^{1}$)3 and (*S*R$2_{2^{p}}^{1}$)4 scheme.

When operating near the nominal nutation frequency, all (*S*R$2_{2^{p}}^{1}$)*M* sequences provide efficient magnetization transfers among spins with equal chemical shifts ($\Delta_{\mathrm{iso}}=0$); see Figure 4a,b. For such cases, the (*S*R2${}_{2}^{1}$)3 and (*S*R2${}_{2}^{1}$)4 schemes perform better than than their more complex analogs. Nonetheless, the magnetization transfers obtained from the (*S*R2${}_{2}^{1}$)*M* sequences become quenched even for moderately large $\Delta_{\mathrm{iso}}$-values and minor deviations from the nominal nutation frequency $\nu_{\mathrm{nut}}^{\mathrm{CT}}=12$ kHz. While the largest magnetization-transfer efficiency deteriorates for increasing $\Delta_{\mathrm{iso}}$ for all recoupling schemes, the compensation to rf-amplitude mis-settings (and thereby to rf inhomogeneity) of the (*S*R2${}_{4}^{1}$)*M* and (*S*R2${}_{8}^{1}$)*M* members are markedly better than for the (*S*R2${}_{2}^{1}$)*M* counterparts. Also, for a growing chemical-shift dispersion, the relative advantage of the (*S*R2${}_{8}^{1}$)*M* schemes become more pronounced relative to their (*S*R2${}_{4}^{1}$)*M* analogs. Indeed, as discussed in refs. [57,58,76], the robustness to the *combined* effects of resonance offsets and rf-amplitude errors improves at each recursive pulse-sequence expansion step (increasing *p* of *S*R$2_{2^{p}}^{1}$). The improved performance of the *S*R2${}_{4}^{1}$ scheme relative to *S*R2${}_{2}^{1}$ was reported earlier in the context of 2QF applications to half-integer spins [49,76]. Here, we show that these relative merits also apply to the (*S*R2${}_{2}^{1}$)*M* and (*S*R2${}_{4}^{1}$)*M* schemes when used for magnetization transfers in 1Q–1Q correlation NMR experiments.

For the (*S*R2${}_{4}^{1}$)*M* and (*S*R2${}_{8}^{1}$)*M* recoupling schemes, Figure 4 manifests transfer-efficiency profiles that are somewhat skewed in that the highest efficiencies are normally *not* observed at the nominal nutation frequency $\nu_{\mathrm{nut}}^{\mathrm{CT}}=12$ kHz (i.e., for a relative nutation frequency of 1.0). This is particularly evident for the (*S*R2${}_{4}^{1}$)*M* schemes that reveal the best performance in the range of relative nutation frequencies of 0.85–0.90 (see Figure 4), while their performance for $\nu_{\mathrm{nut}}^{\mathrm{CT}}>12$ kHz deteriorates rapidly for increasing $\nu_{\mathrm{nut}}^{\mathrm{CT}}/\nu_{\mathrm{nut}}^{\mathrm{CT}}($nom) [except for (*S*R2${}_{4}^{1}$)3 in (c)]. This feature accentuates for $\Delta_{\mathrm{iso}}$-values, and may be understood from the dependence of the effective CT-nutation frequency according to $\nu_{\mathrm{nut}}^{\mathrm{CT}}($eff$)=\sqrt{{\left(\nu_{\mathrm{nut}}^{\mathrm{CT}}\right)}^{2}+\Delta_{\mathrm{iso}}^{2}}$ [26]. Hence, for increasing $\Delta_{\mathrm{iso}}$, lower values of $\nu_{\mathrm{nut}}^{\mathrm{CT}}$ satisfy the condition $\nu_{\mathrm{nut}}^{\mathrm{CT}}($eff$)=\nu_{\mathrm{nut}}^{\mathrm{CT}}($nom). These effects are much less pronounced for the (*S*R2${}_{8}^{1}$)*M* sequences, owing to their improved compensation to variations in $\Delta_{\mathrm{iso}}$ (and Ω).

### 4.2. 2D Correlation ${}^{11}$B NMR Experiments on the NCBS Glass

#### 4.2.1. Introduction to the NCBS Glass Structure

**Figure 5:**
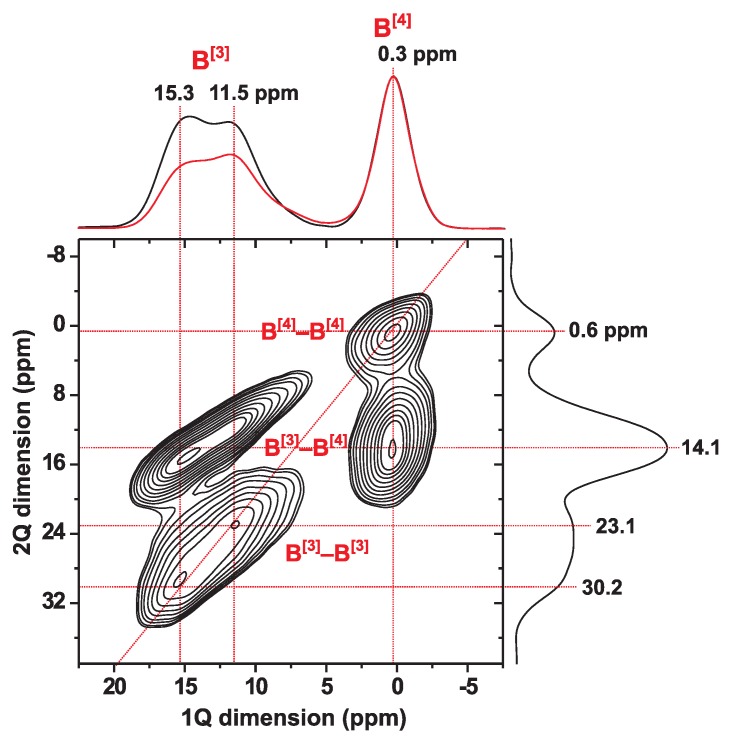
2Q–1Q correlation ${}^{11}$B MAS NMR spectrum recorded from the NCBS glass at $B_{0}=14.1$ T and 24.0 kHz MAS, using one completed [*S*R2${}_{2}^{1}$] sequence for 2QC excitation and reconversion ($\tau_{\mathrm{exc}}=\tau_{\mathrm{rec}}=167$ μs). The 2D NMR spectrum reveals 2Q–1Q correlations from B${}^{\left[3\right]}$–O–B${}^{\left[3\right]}$, B${}^{\left[3\right]}$–O–B${}^{\left[4\right]}$, as well as B${}^{\left[4\right]}$–O–B${}^{\left[4\right]}$ linkages in the borosilicate glass network. Projections along the 2Q and 1Q dimensions are shown to the right and at the top of the 2D NMR spectrum, respectively, together with the MAS NMR spectrum acquired directly by single pulses (red trace).

The NCBS glass structure consists of SiO${}_{4}$ and BO${}_{4}$ tetrahedra (B${}^{\left[4\right]}$ coordinations) along with planar BO${}_{3}$ (B${}^{\left[3\right]}$ coordination) groups, which are interlinked to form a borosilicate network [56,93]. This glass is nominally free from non-bridging oxygen (NBO; O${}^{-}$) species, where NMR indicated 3% of NBO out of the O population [56]. Hence, essentially all of the Na${}^{+}$ and Ca${}^{2+}$ cations balance the negative charges of the [BO${}_{4}$]${}^{-}$ groups. In analogy with the [AlO${}_{4}$]${}^{-}$ tetrahedra in crystalline and amorphous aluminosilicate phases [10,94,95], the negatively charged [BO${}_{4}$]${}^{-}$ moieties have generally been assumed not to form direct linkages (B${}^{\left[4\right]}$–O–B${}^{\left[4\right]}$) in borate/borosilicate glasses [96,97], disregarding B-rich borosilicate glass compositions for which B${}^{\left[4\right]}$–O–B${}^{\left[4\right]}$ bridges cannot be avoided, owing to a high fractional population of the BO${}_{4}$ groups and/or a high NBO content of the glass; see Equation (1) of ref. [56]. Yet, recently Yu et al. [56] provided unambiguous experimental evidence that B${}^{\left[4\right]}$–O–B${}^{\left[4\right]}$ are abundant motifs in Na and mixed-Na/Ca based borosilicate glasses over large compositional spaces (we also note that aluminosilicate glasses comprising trivalent rare-earth ions revel an essentially random Al/Si intermixing that implies substantial amounts of Al${}^{\left[4\right]}$–O–Al${}^{\left[4\right]}$ bridges [10,54]). The presence of B${}^{\left[4\right]}$–O–B${}^{\left[4\right]}$ bonding was established by 2Q–1Q correlation ${}^{11}$B MAS NMR experiments using one completed [*S*R2${}_{2}^{1}$] sequence for 2QC excitation [56], such as that obtained from the NCBS glass and shown in Figure 5.

The ${}^{11}$B MAS NMR spectrum shown in Figure 5 reveals two main resonances: one narrow from the symmetric ${}^{11}$BO${}_{4}$ tetrahedra and one broad from the planar ${}^{11}$BO${}_{3}$ groups. The respective ${}^{11}$B${}^{\left[4\right]}$ and ${}^{11}$B${}^{\left[3\right]}$ sites are associated with average quadrupolar products ${\bar{C}}_{Q\eta }^{\left[4\right]}=0.44$ MHz and ${\bar{C}}_{Q\eta }^{\left[3\right]}=2.67$ MHz, respectively. The second-order quadrupolar broadening of the ${}^{11}$B${}^{\left[3\right]}$ NMR signals produce 2Q–1Q correlation “ridges” that extend along both dimensions of the 2D NMR spectrum (Figure 5), where the 2QC shift is the sum of each $\delta_{B}^{\left[3\right]}$ and $\delta_{B}^{\left[4\right]}$ shift of the respective correlated 1Q ${}^{11}$B${}^{\left[3\right]}$ and ${}^{11}$B${}^{\left[4\right]}$ shifts. The 2Q–1Q correlation ${}^{11}$B NMR spectrum in Figure 5 gives evidence that all three B${}^{\left[3\right]}$–O–B${}^{\left[3\right]}$, B${}^{\left[4\right]}$–O–B${}^{\left[4\right]}$, and B${}^{\left[3\right]}$–O–B${}^{\left[4\right]}$ linkages are present, with the latter dominating in the NCBS glass networks. The ${}^{11}$B–${}^{11}$B dipolar coupling constants in borosilicate glasses range between 700–900 Hz, where those of ${}^{11}$B${}^{\left[3\right]}$–${}^{11}$B${}^{\left[3\right]}$ and ${}^{11}$B${}^{\left[4\right]}$–${}^{11}$B${}^{\left[4\right]}$ are at the higher and lower end, respectively [56]. Analysis of the 2D NMR spectrum revealed the set of fractional populations ($x_{B}^{pq}$) of B${}^{\left[p\right]}$–O–B${}^{\left[q\right]}$ linkages $\{x_{B}^{33}$, $x_{B}^{34}$, $x_{B}^{44}\}=\{0.26$, 0.58, 0.16}, implying that 26% directly connected BO${}_{4}$ tetrahedra account for all of B–O–B bridges of the NCBS glass structure; see ref. [56] for details.

#### 4.2.2. 1Q–1Q Correlation ${}^{11}$B NMR Results

**Figure 6:**
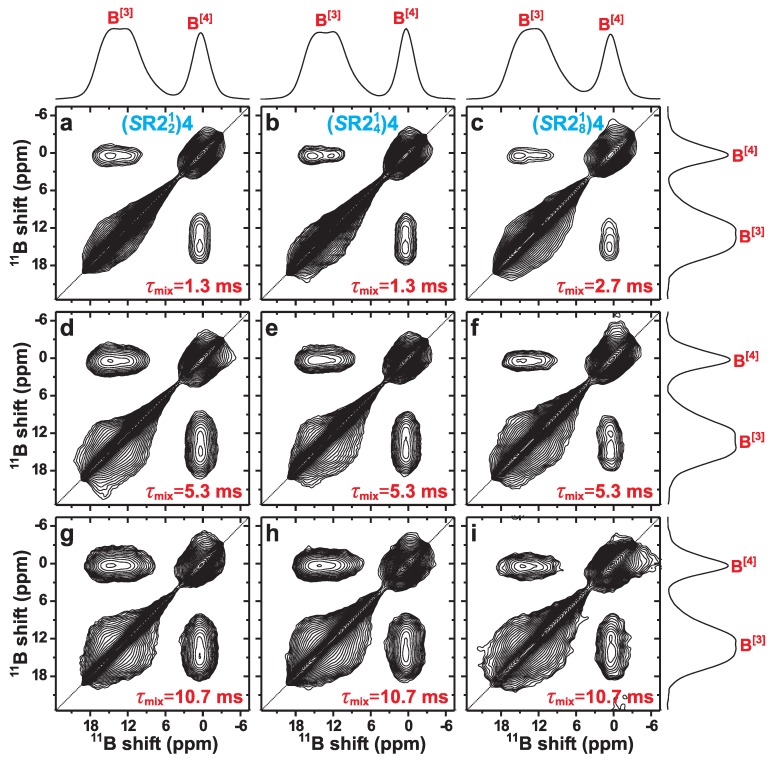
1Q–1Q correlation ${}^{11}$B NMR spectra obtained from the NCBS glass ($B_{0}=14.1$ T, $\nu_{r}=24.00$ kHz) by using the rf-pulse scheme of Figure 1, and employing the (*S*R2${}_{2}^{1}$)4 (left panel), (*S*R2${}_{4}^{1}$)4 (mid panel) and (*S*R2${}_{8}^{1}$)4 (right panel) recoupling schemes during mixing periods ($\tau_{\mathrm{mix}}$) of (**a**,**b**) 1.33 ms, (**c**) 2.67 ms, (**d**–**f**) 5.33 ms, and (**g**–**i**) 10.67 ms. Projections along the horizontal and vertical spectral dimensions are shown at the top and to the right, respectively [only for the spectra in (**a**–**c**,**f**,**i**)].

Figure 6 displays 1Q–1Q correlation ${}^{11}$B MAS NMR spectra recorded by using the (*S*R2${}_{2}^{1}$)4 (left panel), (*S*R2${}_{4}^{1}$)4 (mid panel), and (*S*R2${}_{8}^{1}$)4 (right panel) dipolar recoupling sequences during progressively longer mixing intervals. As highlighted previously in the context of NMR correlation experiments of half-integer spins [8,38,41], 1Q–1Q correlation NMR experimentation may only unambiguously establish proximities among *distinct* sites in an inorganic network structure, i.e., the B${}^{\left[3\right]}$–O–B${}^{\left[4\right]}$ linkages for the present case of the NCBS glass. Nonetheless, they account for 58% of all B${}^{\left[p\right]}$–O–B${}^{\left[q\right]}$ bridges (see Section 4.2.1). These signals appear as a pair of cross peaks connecting the two diagonal peaks associated with each ${}^{11}$B${}^{\left[3\right]}$ and ${}^{11}$B${}^{\left[4\right]}$ resonance [8,9,30]. The ${}^{11}$B${}^{\left[3\right]}$–${}^{11}$B${}^{\left[3\right]}$ and ${}^{11}$B${}^{\left[4\right]}$–${}^{11}$B${}^{\left[4\right]}$ “auto-correlation” peaks from the respective ${}^{11}$B${}^{\left[3\right]}$→${}^{11}$B${}^{\left[3\right]}$ and ${}^{11}$B${}^{\left[4\right]}$→${}^{11}$B${}^{\left[4\right]}$ magnetization transfers overlap with the strong ${}^{11}$B${}^{\left[3\right]}$ and ${}^{11}$B${}^{\left[4\right]}$ NMR signals from non-exchanged magnetization along the diagonal. Unfortunately, this makes the proof of spatial proximities among “like” ${}^{11}$B${}^{\left[p\right]}$ sites ambiguous. In Figure 6, the identification of direct B${}^{\left[3\right]}$–O–B${}^{\left[3\right]}$ and B${}^{\left[4\right]}$–O–B${}^{\left[4\right]}$ structural fragments are hinted by a diffuse broadening of the respective NMR peaks along the diagonal, as may be verified from the 2D NMR spectra that were recorded for increasing mixing periods. Such signal-broadening effects of the ${}^{11}$B autocorrelation signals from borosilicate glasses were discussed further by Murakami et al. [69], and by Edén and Frydman [17] in the context of vitreous B${}_{2}$O${}_{3}$.

**Figure 7:**
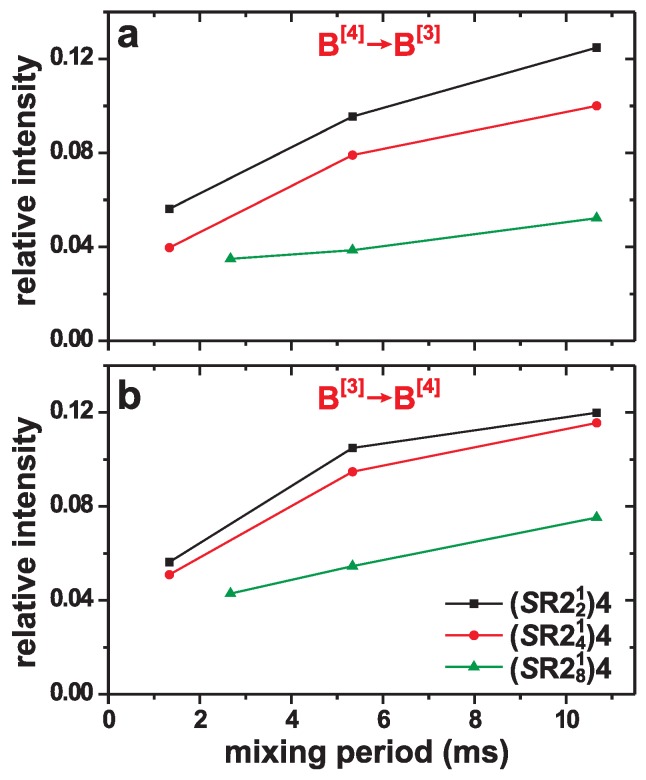
Relative integrated 2D NMR cross-peak intensities of the 1Q–1Q NMR spectra in Figure 6 plotted against the mixing period for the (**a**) ${}^{11}$B${}^{\left[4\right]}\to^{11}$B${}^{\left[3\right]}$ (upper left cross peak) and (**b**) ${}^{11}$B${}^{\left[3\right]}\to^{11}$B${}^{\left[4\right]}$ (lower right cross peak) magnetization transfers observed using either the (*S*R2${}_{2}^{1}$)4, (*S*R2${}_{4}^{1}$)4, or (*S*R2${}_{8}^{1}$)4 schemes. The sum of integrated intensities are normalized to unity for each 2D NMR spectrum, such that each plotted data-point represents the fraction of the total 2D NMR intensity for the respective mixing period and recoupling sequence.

We next focus on the unambiguously evidenced B${}^{\left[3\right]}$–O–B${}^{\left[4\right]}$ linkages associated with the cross-peak ridges observed in Figure 6. Regardless of which (*S*R$2_{2^{p}}^{1})$4 recoupling scheme is applied during the mixing period, the ${}^{11}$B NMR cross-peak intensity grows. Yet, at a fixed value of $\tau_{\mathrm{mix}}$, the (*S*R2${}_{2}^{1}$)4 scheme produces stronger correlation signals than its (*S*R2${}_{4}^{1}$)4 and (*S*R2${}_{8}^{1}$)4 counterparts. The overall trend of improved magnetization exchange along the series of recoupling schemes, (*S*R2${}_{8}^{1}$)4 < (*S*R2${}_{4}^{1}$)4 < (*S*R2${}_{2}^{1}$)4, is most apparent in Figure 7 that contrasts the integrated 2D NMR-signal intensities of the two cross peaks in each spectrum of Figure 6. Notably, we experimentally observed similar 2D NMR signal-intensity oscillations against the resonance offset as in the simulations of Figure 3. The 2D NMR spectra shown for the (*S*R2${}_{4}^{1}$)4 and (*S*R2${}_{8}^{1}$)4 schemes in Figure 6 were selected from the best results of 2D NMR acquisitions using two distinct rf-carrier frequencies (i.e., resonance-offset values), where the resulting NMR intensities varied by 20-30% among the two frequency values. Because the most/least favorable offset is not *a priori* known, it is not possible to arrange precise comparisons among the three (*S*R$2_{2^{p}}^{1})$4 schemes at their respective optimum conditions.

#### 4.2.3. Relative Merits of the (*S*R$2_{2^{p}}^{1})M$ Recoupling Schemes

We conclude that while *any* (*S*R$2_{2^{p}}^{1}$)4 dipolar recoupling sequence give unambiguous evidence for ${}^{11}$B${}^{\left[3\right]}$–O–${}^{11}$B${}^{\left[4\right]}$ linkages in the NCBS glass network for mixing intervals $\tau_{\mathrm{mix}}⩾1.3$ ms (Figure 6), the M=4 MQ-phase cycle based on the simplest *S*R2${}_{2}^{1}$ scheme performed best. In the case of the NCBS glass, no advantages are offered by the more complex (*S*R2${}_{4}^{1}$)4 and (*S*R2${}_{8}^{1}$)4 supercycles, where the (*S*R2${}_{8}^{1}$)4 option gives a significantly worse NMR-signal sensitivity and magnetization transfers as compared with (*S*R2${}_{2}^{1}$)4 (see Figure 6 and Figure 7). Considering the simulation results of Figure 3, the experimentally observed relative merits of the three (*S*R$2_{2^{p}}^{1}$)4 recoupling schemes are rather surprising. Yet, the chemical-shift separation between the ${}^{11}$B${}^{\left[3\right]}$ and ${}^{11}$B${}^{\left[4\right]}$ sites is relatively small (≈17 ppm, i.e., 3.3 kHz), where a good performance of *S*R2${}_{2}^{1}$-based schemes are indeed reported in previous 2QF and 2Q–1Q correlation NMR evaluations for similar cases where resonance offsets are low or absent [38,51,52,53,55,56]. Experiments reveal that the (*S*R2${}_{4}^{1}$)*M* and (*S*R2${}_{8}^{1}$)*M* schemes give significantly better magnetization *only* for scenarios of (moderately) large chemical-shift differences, for which the (*S*R2${}_{2}^{1}$)*M* counterparts perform poorly.

In the following, we consider the relative NMR-signal *sensitivities* (rather than the magnetization transfer efficiencies) offered by the various (*S*R$2_{2^{p}}^{1}$)*M* and (*S*R$4_{4}^{1}$)*M* dipolar recoupling schemes. Notably, the ${}^{11}$B NMR-signal intensities observed from the NCBS glass when employing the present (*S*R2${}_{2}^{1}$)Mand (*S*R2${}_{4}^{1}$)*M* sequences are markedly better then those of the windowed (*S*R$4_{4}^{1}$)*M* recoupling options utilized by Edén et al. [30] (see Section 2): relative to the integrated 2D NMR-signal intensity obtained from the (*S*R2${}_{2}^{1}$)4 scheme in Figure 6g, only 41% and 6% was observed when applying the (*S*R$4_{4}^{1}$)3 or (*S*R$4_{4}^{1}$)4 sequences with $f_{180}=0.30$ (see Section 2) for a mixing period of $\tau_{\mathrm{mix}}=10.7$ ms, respectively (as obtained by recording a 1D NMR spectrum according to the protocol of Figure 1 with $t_{1}=0$; data not shown). Next considering the NMR-signal intensity obtained among the various (*S*R$2_{2^{p}}^{1}$)*M*sequences and again monitoring the fractional intensity relative to the 2D NMR spectrum of Figure 6g, the set of {(*S*R2${}_{2}^{1}$)4, (*S*R2${}_{4}^{1}$)4, (*S*R2${}_{8}^{1}$)4} schemes revealed a {100%, 57%, 28%} signal retention, respectively, whereas the corresponding numbers are {112%, 55%, 21%} when employing the {(*S*R2${}_{2}^{1}$)3, (*S*R2${}_{4}^{1}$)3, (*S*R2${}_{8}^{1}$)3} supercycles (data not shown). Hence, except for the (*S*R2${}_{8}^{1}$)*M* supercycles, the (*S*R$2_{2^{p}}^{1}$)*M*, recoupling schemes offer markedly better S/N than the windowed (*S*R$4_{4}^{1}$)*M* counterparts of ref. [30].

The low-power (*S*R$2_{2^{p}}^{1}$)*M* rf-pulse trains are particularly advantageous for recoupling spin sites with low quadrupolar coupling constants/products, such as the ${}^{11}$B${}^{\left[4\right]}$ nuclei in the NCBS glass (and the ${}^{27}$Al${}^{\left[6\right]}$ sites of AlPO-CJ19; see Section 4.3). All (*S*R$2_{2^{p}}^{1}$)*M* schemes retain similar NMR-signal fractions of 0.32–0.38 from the ${}^{11}$B${}^{\left[4\right]}$ sites in the 2D NMR spectrum for the mixing period of 10.7 ms, in excellent agreement with the “apparent” fractional population $x_{B}^{\left[4\right]}=0.36$ of the NCBS structure (see Section 3.2.1). In contrast, the corresponding integrated ${}^{11}$B${}^{\left[4\right]}$ NMR signal fraction obtained from the (*S*R4${}_{4}^{1}$)3 and (*S*R4${}_{4}^{1}$)4 schemes with $f_{180}=0.30$ only amounted to 0.20 and 0.13, respectively. Hence, only a few percent of the initial ${}^{11}$B${}^{\left[4\right]}$ magnetization remains after application of the (*S*R$4_{4}^{1}$)*M* schemes that involve stronger rf pulses. These losses accentuate for prolonged mixing periods and/or for stronger 180${}^{\circ }$ recoupling pulses.

Concerning the merits of the M=3 supercycles relative to their M=4 counterparts, often (but not always), we observe experimentally that for a given dipolar recoupling sequence S of the (S)*M* supercycle, the NMR-signal strength is slightly higher for the M=3 scheme as compared with its M=4 counterpart. However, these effects appear to be spin-system-dependent (i.e., sample dependent), where for instance the ${}^{11}$B NMR experiments on the NCBS glass manifest even slightly higher NMR-signal intensities from the (*S*R$2_{2^{p}}^{1}$)4schemes than those of (*S*R$2_{2^{p}}^{1}$)3 (see above). In contrast, the windowed (*S*R$4_{4}^{1}$)4 supercycle yields much higher NMR-signal losses than its (*S*R$4_{4}^{1}$)3 analog. The reasons for these observations are unknown, but a more comprehensive evaluation of the various (*S*R$2_{2^{p}}^{1}$)*M* and (*S*R$4_{4}^{1}$)*M* recoupling options is in progress and will be published elsewhere.

### 4.3. ${}^{27}$Al Correlation Experiments on AlPO-CJ19

**Figure 8:**
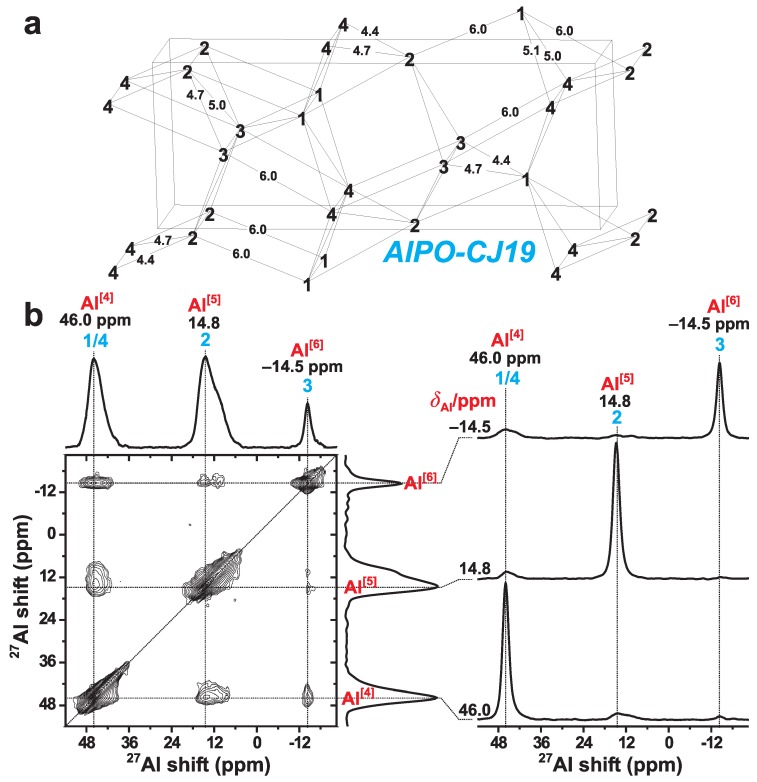
(**a**) Structural fragment of AlPO-CJ19 [72]. Only the four Al sites (Al**1-**Al**4**) are indicated, along with the shortest Al*j*–Al*k* interatomic distances (numbers in Å) among inequivalent sites (j≠k). All shortest Al*j*–Al*j* distances between equivalent sites (j=1,2,3,4) are around 5.1 Å. The Al**1**, Al**2**, Al**3**, and Al**4** sites are identified with their respective Al${}^{\left[4\right]}$, Al${}^{\left[5\right]}$, and Al${}^{\left[6\right]}$ coordinations in the legend. (**b**) 1Q–1Q correlation ${}^{27}$Al MAS NMR spectrum recorded from AlPO-CJ19 ($B_{0}=14.1$ T, $\nu_{r}=24.0$ kHz), using the (*S*R2${}_{4}^{1}$)3 sequence for magnetization transfers during a mixing period of $\tau_{\mathrm{mix}}=30.0$ ms. Slices extracted at the as-indicated shifts along the vertical spectral dimensions are shown in the right panel.

We next consider the challenging case of spin-5/2 in the context of ${}^{27}$Al recoupling in the open framework aluminophosphate AlPO-CJ19. A structural fragment is depicted in Figure 8a, where each of the four crystallographically inequivalent Al sites is labelled by a numbers **1**–**4**) and assigned to its respective Al${}^{\left[4\right]}$, Al${}^{\left[5\right]}$, and Al${}^{\left[6\right]}$ coordination in (b) [72]. The AlPO-CJ19 structure is built from Al–O–P–O–Al motifs where Al and P alternate strictly between the O bridges [72], as confirmed from 2Q–1Q ${}^{27}$Al NMR experiments [38,49]. Consequently, the closest ${}^{27}$Al–${}^{27}$Al neighbors are separated by four bonds, which yields the range 0.44–0.6 nm of shortest distances (Figure 8a), and thereby to comparably small dipolar coupling constants between 40–100 Hz. Over a radius of 0.6 nm, *every* Al*j* site (j=1, **2**, **3**, **4**) is connected to *all* others (Al**1**, Al**2,**Al**3**, and Al**4**). The overall closest Al*j*–Al*k* (dipolar-coupling) contacts are observed for the Al${}^{\left[4\right]}$**1**–Al${}^{\left[6\right]}$**3** and Al${}^{\left[5\right]}$**2**–Al${}^{\left[4\right]}$**4** pairs, whose two shortest distances are both ≈0.44 nm and ≈0.47 nm. On the other hand, each shortest Al${}^{\left[p\right]}j$–Al${}^{\left[p\right]}j$ distance involving equivalent sites (j=1,2, 3, 4) is around 0.51 nm, whereas those of the Al${}^{\left[4\right]}$**1**–Al${}^{\left[5\right]}$**2** and Al${}^{\left[6\right]}$**3**–Al${}^{\left[4\right]}$**4** are markedly longer (≈0.6 nm); see Figure 8a.

Figure 8b shows the 1Q–1Q correlation ${}^{27}$Al NMR spectrum obtained by applying the (*S*R2${}_{4}^{1}$)3 recoupling scheme for $\tau_{\mathrm{mix}}=30$ ms at $B_{0}=14.1$ T and $\nu_{r}=24.0$ kHz. At this magnetic field, the AlPO-CJ19 sample is a challenging case for ${}^{27}$Al–${}^{27}$Al recoupling: owing to the small dipolar interactions, the comparatively slow magnetization transfers must proceed for a long mixing period (compare with the $\tau_{\mathrm{mix}}$-values employed for the NCBS glass in Figure 6, which exhibit more than one order of magnitude larger ${}^{11}$B–${}^{11}$B interactions), while the substantial chemical-shift dispersion among the three ${}^{27}$Al${}^{\left[p\right]}$ sites produces a large range of resonance offsets at $B_{0}=14.1$ T. The isotropic chemical shifts of the four ${}^{27}$Al sites are –13.4 ppm (Al${}^{\left[6\right]}3$), 17.0 ppm (Al${}^{\left[5\right]}2$), 47.6 ppm (Al${}^{\left[4\right]}1$), and 48.1 ppm (Al${}^{\left[4\right]}4$) [72]. This leads to sizable shift-difference of $\Delta_{\mathrm{iso}}=$9.62 kHz between the ${}^{27}$Al${}^{\left[4\right]}$ and ${}^{27}$Al${}^{\left[6\right]}$ coordinations, whereas those for the ${}^{27}$Al${}^{\left[4\right]}$–${}^{27}$Al${}^{\left[5\right]}$ and ${}^{27}$Al${}^{\left[5\right]}$–${}^{27}$Al${}^{\left[6\right]}$ pairs are both around 4.8 kHz. Note that the very similar chemical shifts of the two ${}^{27}$Al${}^{\left[4\right]}1$ and ${}^{27}$Al${}^{\left[4\right]}4$ leave their resonances unresolved in Figure 8b. Further complications for efficient dipolar recoupling arise from the widely differing quadrupolar coupling constants, where the quadrupolar products ${\bar{C}}_{Q\eta }^{\left[p\right]}$ of the {Al${}^{\left[4\right]}1$, Al${}^{\left[4\right]}4$, Al${}^{\left[5\right]}2$, Al${}^{\left[6\right]}3$} are {3.8, 2.6, 4.2, 1.4} MHz [72]. Here we did not attempt any (*S*R2${}_{2}^{1}$)*M* implementation, because previous 2Q–1Q correlation NMR experimentation at the lower magnetic field of $B_{0}=9.4$ T (and thereby lower $\Delta_{\mathrm{iso}}$-values) has already demonstrated the superiority of the [*S*R2${}_{4}^{1}$] scheme over [*S*R2${}_{2}^{1}$] [38,49].

Given these obstacles, it is gratifying that the 1Q–1Q correlation ${}^{27}$Al NMR spectrum in Figure 8b that utilized the (*S*R2${}_{4}^{1}$)3 supercycle reveals cross peaks among each of the three ${}^{27}$Al${}^{\left[p\right]}$–${}^{27}$Al${}^{\left[q\right]}$ pairs of distinct Al coordinations. Consequently, despite that all cross peaks are relatively weak, the results of Figure 8b evidence that the (*S*R2${}_{4}^{1}$)3 scheme enables magnetization transfers among all Al*j*–Al*k* pairs in AlPO-CJ19 even at the moderately high field of 14.1 T. The strongest NMR correlations are observed for the ${}^{27}$Al${}^{\left[4\right]}$–${}^{27}$Al${}^{\left[5\right]}$ pair, which comprises (unresolved) signal contributions from both the ${}^{27}$Al${}^{\left[4\right]}1$ and ${}^{27}$Al${}^{\left[4\right]}4$ sites. This result is consistent with the overall strongest (dipolar coupling) contact for Al${}^{\left[5\right]}$**2**–Al${}^{\left[4\right]}$**4**. The ${}^{27}$Al${}^{\left[4\right]}$–${}^{27}$Al${}^{\left[6\right]}$ pair reveals the second most intense cross peaks in the 2D NMR spectrum; although the Al${}^{\left[4\right]}$**1**–Al${}^{\left[6\right]}$**3** pair exhibit essentially identical shortest distances (as commented above), the slightly lower intensities of the cross peaks stemming from the ${}^{27}$Al${}^{\left[4\right]}$–${}^{27}$Al${}^{\left[6\right]}$ pair relative to those of ${}^{27}$Al${}^{\left[4\right]}$–${}^{27}$Al${}^{\left[5\right]}$ may be traced to some interferences in the magnetization transfers from the twice as large chemical-shift separation among the ${}^{27}$Al${}^{\left[4\right]}$ and ${}^{27}$Al${}^{\left[6\right]}$ sites. Nonetheless, these effects are minor. Moreover, smaller 2D NMR cross peaks are observed for the ${}^{27}$Al${}^{\left[5\right]}$–${}^{27}$Al${}^{\left[6\right]}$ pair, as is expected from the weaker Al${}^{\left[5\right]}$**2**–Al${}^{\left[6\right]}$**3** contacts, whose two shortest distances are 0.47 nm and 0.50 nm, respectively [72]. A further reduction of the cross peak intensities may stem from the sizable difference of quadrupolar products between the ${}^{27}$Al${}^{\left[5\right]}$ (${\bar{C}}_{Q\eta }^{\left[5\right]}=4.2$ MHz) and ${}^{27}$Al${}^{\left[6\right]}$ (${\bar{C}}_{Q\eta }^{\left[5\right]}=1.4$ MHz) sites.

We conclude that the 1Q–1Q correlation ${}^{27}$Al NMR results obtained using the (*S*R2${}_{4}^{1}$)3 recoupling sequence for magnetization transfers accord well with the various ${}^{27}$Al–${}^{27}$Al distances of the AlPO-CJ19 structure (Figure 8a).

## 5. Conclusions

We have explored MQ-phase cycles of the *S*R$2_{2^{p}}^{1}$ family of homonuclear dipolar recoupling sequences for driving longitudinal magnetization transfers among half-integer spin quadrupolar nuclei undergoing fast MAS (20–30 kHz) at a moderately high magnetic field of 14.1 T. These (*S*R2${}_{2}^{1}$)*M*, (*S*R2${}_{4}^{1}$)*M*, and (*S*R2${}_{8}^{1}$)*M* recoupling schemes with M=3 and M=4 were utilized in 1Q–1Q correlation NMR experiments applied in the contexts of ${}^{11}$B NMR on a borosilicate glass (NCBS) and ${}^{27}$Al NMR on the open framework aluminophosphate AlPO-CJ19. Numerical simulations of pairs of dipolar-coupled spins-3/2 revealed a progressively improved stability of the magnetization transfers for variations in resonance offsets and rf-amplitude errors of the recoupling pulses along the series (*S*R2${}_{2}^{1}$)*M*<(*S*R2${}_{4}^{1}$)*M*<(*S*R2${}_{8}^{1}$)*M*, in agreement with previous findings from related 2Q–1Q correlation NMR applications of the [*S*R2${}_{2}^{1}$] and [*S*R2${}_{4}^{1}$] schemes to quadrupolar nuclei [49,76].

For dipolar recoupling applications where the chemical-shift dispersion is relatively low ($\Delta_{\mathrm{iso}}≲$3 kHz), we recommend using the simplest (*S*R2${}_{2}^{1}$)3 and (*S*R2${}_{2}^{1}$)4 schemes, which outperformed the more complex (*S*R2${}_{4}^{1}$)*M* and (*S*R2${}_{8}^{1}$)*M* recoupling options for magnetization exchange among the ${}^{11}$B${}^{\left[3\right]}$ and ${}^{11}$B${}^{\left[4\right]}$ sites in the NCBS glass (which feature $\Delta_{\mathrm{iso}}\approx$3 kHz). In such cases of low resonance spreads, no advantages are offered by the (*S*R2${}_{4}^{1}$)*M* and (*S*R2${}_{8}^{1}$)*M* supercycles with M={3, 4}. In contrast, for dipolar recoupling scenarios manifesting a substantial chemical-shift dispersion, such as the ${}^{27}$Al${}^{\left[4\right]}$, ${}^{27}$Al${}^{\left[5\right]}$, and ${}^{27}$Al${}^{\left[6\right]}$ sites in AlPO-CJ19 at 14.1 T, we recommend using either of the (*S*R2${}_{4}^{1}$)*M* or (*S*R2${}_{8}^{1}$)*M* schemes. Yet, our experimental evaluations thus far do not reproduce the superiority of the new (*S*R2${}_{8}^{1}$)3 and (*S*R2${}_{8}^{1}$)4 schemes predicted by the numerical simulations.

Concerning the relative merits of the M=3 supercycles relative to their M=4 counterparts, we often (but not always) observe experimentally that for a given dipolar recoupling sequence S of the (S)*M* supercycle, the NMR-signal strength is slightly higher for the M=3 supercycle relative to its M=4 counterpart. Yet, the precise responses of the M={3, 4} supercycles appear to depend both on the sample and particular pulse sequence. Moreover, numerical simulations (e.g., see Figure 3 and Figure 4) do not indicate any significant advantage of either option. We found that the M=3 and M=4 MQ-phase cycle options gave similar results when combined with any *S*R$2_{2^{p}}^{1}$ scheme and employed during the mixing segment in 1Q–1Q correlation NMR experiments on the NCBS glass. For the windowed (*S*R$4_{4}^{1}$)*M* schemes of ref. [30], on the other hand, much higher NMR-signal losses resulted when using the (*S*R$4_{4}^{1}$)4 scheme relative to (*S*R$4_{4}^{1}$)3. Furthermore, *both* (*S*R$4_{4}^{1}$)*M* recoupling schemes yielded overall larger NMR-signal losses than their (*S*R$2_{2}^{1}$)*M* and (*S*R$2_{4}^{1}$)*M* counterparts. Here the small (average) quadrupolar product of the ${}^{11}$B${}^{\left[4\right]}$ sites (${\bar{C}}_{Q\eta }^{\left[4\right]}=0.44$ MHz) severely compromises its NMR-signal sensitivity obtained when utilizing the (*S*R$4_{4}^{1}$)*M* schemes, which involves stronger $180^{\circ }$ pulses and thereby higher CT-magnetization losses from the ${}^{11}$B${}^{\left[4\right]}$ sites than when using the (*S*R$2_{2^{p}}^{1}$)*M* supercycles. In contrast, the latter recoupling sequences preserved each relative ${}^{11}$B${}^{\left[3\right]}$ and ${}^{11}$B${}^{\left[4\right]}$ NMR-signal intensity according to that of the respective site population of the NCBS glass, which is a decisive advantage.

A comprehensive experimental and numerical evaluation of the (*S*R$2_{2^{p}}^{1}$)*M* supercycles relative to the (*S*R$4_{4}^{1}$)*M* schemes of ref. [30], as well as to other zero-quantum recoupling options, is underway and will be presented elsewhere.
